# Attaching and Effacing *Escherichia coli* Downregulate DNA Mismatch Repair Protein *In Vitro* and Are Associated with Colorectal Adenocarcinomas in Humans

**DOI:** 10.1371/journal.pone.0005517

**Published:** 2009-05-13

**Authors:** Oliver D. K. Maddocks, Abigail J. Short, Michael S. Donnenberg, Scott Bader, David J. Harrison

**Affiliations:** 1 Division of Pathology, Institute of Genetics and Molecular Medicine, The University of Edinburgh, Western General Hospital, Edinburgh, United Kingdom; 2 Division of Infectious Diseases, University of Maryland School of Medicine, Baltimore, Maryland, United States of America; Technical University Munich, Germany

## Abstract

**Background:**

Mucosa-associated *Escherichia coli* are frequently found in the colonic mucosa of patients with colorectal adenocarcinoma, but rarely in healthy controls. Chronic mucosal *E. coli* infection has therefore been linked to colonic tumourigenesis. *E. coli* strains carrying *eae* (encoding the bacterial adhesion protein intimin) attach intimately to the intestinal mucosa and are classed as attaching and effacing *E. coli* (AEEC). Enteropathogenic *Escherichia coli* (EPEC) are the most common form of AEEC identified in man. EPEC utilise a type III secretion system to translocate effector proteins into host cells and infection induces wide-ranging effects on the host cell proteome. We hypothesised that EPEC infection could influence molecular pathways involved in colorectal tumourigenesis.

**Methodology/Principal Findings:**

When co-cultured with human colorectal cell lines, EPEC dramatically downregulated the expression of key DNA mismatch repair proteins MSH2 and MLH1 in an attachment specific manner. Cytochrome *c* staining and TUNEL analysis confirmed that this effect was not a consequence of apoptosis/necrosis. *Ex vivo* human colonic mucosa was co-cultured with EPEC and probed by immunofluorescence to locate adherent bacteria. EPEC entered 10% of colonic crypts and adhered to crypt epithelial cells, often in the proliferative compartment. Adenocarcinoma and normal colonic mucosa from colorectal cancer patients (n = 20) was probed by immunofluorescence and PCR for AEEC. Mucosa-associated *E. coli* were found on 10/20 (50%) adenocarcinomas and 3/20 (15%) normal mucosa samples (P<0.05). AEEC were detected on 5/20 (25%) adenocarcinomas, but not normal mucosa samples (P<0.05).

**Significance/Conclusions:**

The ability of EPEC to downregulate DNA mismatch repair proteins represents a novel gene-environment interaction that could increase the susceptibility of colonic epithelial cells to mutations and therefore promote colonic tumourigenesis. The potential role of AEEC in colorectal tumourigenesis warrants further investigation.

## Introduction

Colorectal cancer is responsible for nearly half a million deaths annually, and over 940,000 new cases are diagnosed worldwide each year [1]. Despite the characterisation of many aetiologic genetic changes – such as mutation of *APC* and genes encoding DNA mismatch repair proteins – the specific causative factors in the development of sporadic colorectal cancer remain unclear. Gene-environment interactions have a significant influence on the susceptibility to colorectal cancer, but our current understanding of these interactions is limited [2].

Bacteria contribute significantly to the colonic environment and chronic bacterial infection has been linked to colonic tumourigenesis [3]. Studies on cancer patients in the UK and Germany reveal that mucosa-associated bacteria are more frequently identified in colon tissue from patients with adenocarcinomas than in controls [4], [5]. Swidsinski *et al.* report that only 3% of colon mucosa biopsies from asymptomatic controls tested positive for bacteria. In contrast, biopsies from 92% of patients with colonic adenomas or carcinomas held bacteria, with *Escherichia coli* the predominant bacteria in 70% of patients [5]. Similarly, Martin *et al.* found that 70% of colorectal cancer patients had mucosa-associated bacteria, and that a significant proportion of the bacterial isolates were *E. coli* strains that adhered to HT29 cells *in vitro* [4]. These studies clearly demonstrate a link between mucosally adherent *E. coli* and colon cancer. Whether this relationship is a cause or effect of tumourigenesis is an important question that is yet to be resolved.

Bacteria that carry the *eae* gene, which encodes the bacterial adhesion protein intimin, are able to attach intimately to the intestinal epithelium and are classified as attaching and effacing (AE). The *eae* gene is carried by enteropathogenic *E. coli* (EPEC), enterohemorrhagic *E. coli* (EHEC), *E. albertii* and *Citrobacter rodentium* strains, and is not carried by *E. coli* strains characterised as belonging to the normal human microflora [6]. EPEC is the most commonly identified AE bacteria in man and is a major cause of potentially fatal infantile diarrhoea in developing countries [7]. In developed countries the incidence of EPEC induced diarrhoea has declined, consequently routine screening for EPEC is no longer performed [6]. However, recent studies in Europe and Australia have identified EPEC in 2.5–10.9% of healthy children and (in one study) children and adults [8]–[11], suggesting that a significant percentage of the healthy population in developed countries harbour EPEC within their gastrointestinal microflora. The inability to identify certain strains of EPEC in animals supports the conclusion that man is a natural reservoir for EPEC [7]. Although acute EPEC infection is known to cause diarrhoea, the effects of chronic low-level intestinal colonisation are unknown.

A link between AE bacteria and carcinogenesis is provided by *Citrobacter rodentium*, which causes transmissible colonic hyperplasia [12], reduces the latent period in appearance of chemically induced tumours [13], and promotes colonic adenoma formation in mice [14]. Based on such findings it has recently been suggested that signals delivered by AE bacteria might promote cancer by exacerbating defects in oncogenic pathways in human colonic epithelial tissue [15]. The AE process is dependent on the expression of intimin and translocation of bacterial effector proteins into the host cell via a bacterial type-III secretion system. The genes that encode the secretion system, translocator proteins, several effector proteins and intimin are located in the bacterial locus of enterocyte effacement (LEE) pathogenicity island [16]. The EspB protein is secreted by this system and is required for translocation of effector proteins into the host cell. In EPEC that carry mutant *espB*, effector protein translocation and AE do not occur [17]. By the process of intimate attachment EPEC infection induces host cell protein phosphorylation, cytoskeletal rearrangement and signalling pathway activation [18], [19]. Consequently the expression of hundreds of host cell proteins with wide ranging functions are up and down regulated subsequent to infection [20].

We hypothesised that the intracellular effects of EPEC infection could promote susceptibility to cancer. For this reason we investigated the effects of EPEC on the expression of DNA mismatch repair (MMR) proteins. The DNA MMR system maintains genomic stability by correcting DNA base pair mismatches acquired from replication, recombination and chemical damage. Failure of DNA MMR confers a mutator phenotype, leading to the accumulation of thousands of somatic mutations in DNA microsatellite sequences, a state termed microsatellite instability (MSI). Inactivating mutations of the DNA MMR genes *MSH2* and *MLH1* are the causative genetic feature of Hereditary Non-Polyposis Colorectal Cancer and also occur in up to 15% of sporadic colorectal cancers [21]. Another common mode of mismatch repair gene inactivation is DNA methylation. In sporadic colorectal tumours that present with loss of MLH1 expression, it is often *MLH1* promoter hypermethylation that is responsible for gene silencing [22]. It has been suggested that because *MLH1* silencing leads to colorectal cancer characterised by MSI, *MLH1* silencing is likely to be a relatively early event in carcinogenesis [23].

## Materials and Methods

### Ethics Statement

Ethical approval for the use of human tissue was provided by the NHS Lothian Research Ethics Committee. All information pertaining to subjects and all human samples were used in compliance with UK legislation.

### Bacterial strains and culture conditions

Wild-type (wt) EPEC, strain E2348/69 and mutant (mut) EPEC UMD864 [E2348/69 Δ48-759 *espB1*] [17] stocks were maintained on MacConkey agar supplemented with crystal violet, sodium chloride, 0.15% bile and 0.05% nalidixic acid (all Sigma-Aldrich, Steinheim, Germany). Overnight cultures were grown from single colonies inoculated into Luria Bertani (LB) broth and incubated at 37°C in an orbital shaker.

### Cell lines and culture conditions

Human colorectal cancer cell lines HT29 and SW480 were obtained from The European Collection of Cell Cultures (ECACC). Cells were grown in Roswell Park Memorial Institute (RPMI) 1640 media containing L-glutamine 300 g/L (Gibco/Invitrogen, Paisley, UK), supplemented with 5% fetal calf serum (Labtech/Biosera, Sussex, UK) referred to as R5 media. Stock cells were grown as adherent monolayers in 75 cm^2^ cell culture flasks (Corning/Costar, High Wycombe, UK) and maintained at 37°C in a humidified atmosphere of 5% CO_2_ in air.

### In vitro co-culture

Initial co-culture experiments were performed with HT29 cells grown to 90–100% confluency in 75 cm^2^ cell culture flasks. Overnight EPEC cultures were directly inoculated into the cell culture medium 1 in 100 and flasks were incubated at 37°C in a humidified atmosphere of 5% CO_2_ in air. After 3 h, media was removed from flasks, cells were washed three times with RPMI media and fresh R5 media was added. Mutant EPEC infected cells were re-inoculated with overnight mutant EPEC culture to replace non-adherent bacteria lost during the washing process. The wash cycle was repeated every 3 h for a total time-course of 12 h*. Uninfected controls were either maintained for 12 h without washing or were washed every 3 h in the same way as infected cells. Cells that were allowed to recover from infection were initially co-cultured with wild-type EPEC for 9 and 12 h (as described above) then washed three times with RPMI media and treated with R5 media supplemented with penicillin 200,000 u/L, streptomycin 200 mg/L and gentamicin 100 mg/L (all Gibco/Invitrogen, Paisley, UK) for 36–39 h. Cells used for later co-culture experiments were grown in 10 cm diameter cell culture dishes holding glass cover-slips. For these experiments we introduced a bacterial pre-culture step. Prior to co-culture, overnight EPEC cultures were inoculated into warmed R5 media (1 in 50 dilution) and incubated for 1–1.5 h at 37°C to activate the bacteria [24]. The infected R5 media was then added directly to the cells. After co-culture cells adherent to cover-slips were fixed in paraformaldehyde 4% in PBS and used for immunofluorescence, cells adherent to the dish provided protein for western blotting. [*Subsequent to these experiments we noted that a change in fetal calf serum (to a different supplier) affected bacterial growth during co-culture, but that a minor adjustment of the washing schedule yielded the same results.]

### Immunoblot analysis

Whole cell protein extracts were resolved through pre-cast 4–12% gradient/10% Novex-NuPAGE gels (Invitrogen, Paisley, UK) or 10% sodium dodecyl sulphate/polyacrylamide gels and transferred to Hybond P membranes (GE Healthcare Biosciences, Bucks, UK). Protein levels could not be equalised via conventional densitometry due to the presence of bacterial protein in some samples. Therefore, levels were equalised via successive western blots with reference to beta-actin and proliferating cell nuclear antigen (PCNA) band intensities, coumassie blue staining was also used to aid equal protein loading. All antibodies were diluted in tris-buffered saline with Tween-20 0.2% (TBST) containing 5% powdered milk. Anti-MSH2 Ab-2/FE11 (Calbiochem/Merck, Nottingham, UK) 1∶500, anti-MLH1 (BD Biosciences, Oxford, UK) 1∶1000, anti-beta-actin AC-15 (Sigma-Aldrich, Steinheim, Germany) 1∶5000 and Anti-PCNA C-20 (Santa-Cruz Biotechnology, CA) 1∶1000 primary antibodies and peroxidase conjugated secondary antibodies were applied for 30 minutes–2 hours or overnight. Proteins were detected using ECL Plus® chemiluminescence detection system, membranes were exposed to Hyperfilm (GE Healthcare Biosciences, Bucks, UK).

### Immunofluorescence staining of cells

Fixed cells were washed with PBS, permeabilised with Triton-X-100 0.1% in PBS and blocked with BSA 3% and Tween-20 0.2% in PBS for 1 hour. Cells were then washed with Tween-20 0.2% in PBS (PBST) and incubated with primary antibody solutions for 1 h. After washing with PBST, cells were incubated with secondary antibody solutions for 30 minutes. Finally, cells were washed PBST and mounted onto microscope slides with Vectasheid mounting medium with DAPI (Vector Laboratories, Cambridge, UK). All antibodies were diluted in PBST. The primary antibodies used were anti-MSH2 1∶200, anti-MLH1 1∶200, anti-PCNA 1∶500 (as in immunoblots), anti-cytochrome *c* 1∶500, anti-MTCO2 (both Abcam, Cambridge, UK) 1∶1000, Anti-*E. coli* (Europa Bioproducts, Cambridge, UK) 1∶2000. Secondary antibodies used were Alexa-fluor 488 anti-mouse 1∶1000 and Alexa-fluor 594 anti-sheep and anti-rabbit 1∶1000 (all Invitrogen Molecular Probes, OR). Cells undergoing late stage apoptosis/necrosis were identified based on labelling of DNA strand breaks (TUNEL technology) using an *In Situ* Cell Death Detection Kit (Roche Diagnostics, Mannheim, Germany) according to the manufacturers instructions, and visualised via fluorescence microscopy. Percentages of TUNEL positive cells were calculated by counting at least 400 cells from each experimental condition (n = 3). Positive control cells were treated with DNAse (Qiagen, Crawley, UK). Uninfected HT29 cells were treated with etoposide 1 uM (Sigma-Aldrich, Steinheim, Germany) for 2 hours, followed by 6 hours in R5 media, to act as positive controls for apoptosis via cytochrome *c* staining.

### Ex vivo tissue

The tissue co-culture protocol was adapted from published *in vitro* organ culture (IVOC) methods [25]–[27]. After securing written informed consent, full thickness sections of normal transverse colon tissue were removed from three adult patients undergoing colectomy (due to rectal or endometrial cancer) at the Western General Hospital, Edinburgh. The mucosa was separated from the muscularis, washed in sterile saline and transferred to the laboratory in R5 media supplemented with nalidixic acid 50 mg/L. In the laboratory, tissue was washed three times in warmed PBS, cut into approximately 2 cm^2^ pieces and placed (luminal side upwards) onto pieces of metal gauze within central-well tissue culture dishes containing warmed R5 media with nalidixic acid 50 mg/L. Uninfected control tissue was fixed immediately after washing with PBS. Tissue samples were placed in a pressurised chamber gassed with 95% O_2_ 5% CO_2_ and maintained at 37°C. After a 1 h equilibration period tissue that was to be infected received 200 ul of wild-type EPEC culture (overnight culture diluted 1∶50 in R5 media and activated at 37°C for 1–2 h) added directly to the mucosal surface. After 4 h, tissues were removed from culture dishes, washed thoroughly with warmed PBS and placed back into culture dishes with fresh R5 media. This washing process was repeated every 2–3 h. After a total co-culture period of 12 h, tissue was removed from culture, washed three times in PBS and fixed overnight in 4% buffered formalin. Tissue was embedded in paraffin and sections were cut for immunofluorescence staining.

### Adenocarcinoma tissue

Formalin fixed, paraffin embedded tissue sections were supplied by the Department of Histopathology, University of Edinburgh. Sections of normal and adenocarcinoma tissue from 20 colorectal cancer patients were used. Sections were cut to 3 µm for immunofluorescence staining and 20 µm for PCR.

### PCR

Paraffin embedded tissue sections were subjected to proteinase K digestion, then phenol chloroform extraction of DNA followed by ethanol precipitation in the presence of yeast transfer RNA. Primers for *eae* were: forward 5′ GTG ACG ATG GGG ATC GAT and reverse 5′ GGC TCA ATT TGC TGA GAC CAC GGT T (product 110 bp) or 5′ ACG GCT GCC TGA TAA TGT T (150 bp product). PCR cycle conditions: 94°C 2 min, (94°C 30 sec, 58°C 30 sec)×30 cycles.

### Immunofluorescence staining of human tissue

Tissue sections were de-paraffinised with xylene and alcohol. Antigen retrieval was carried out by heating slides in citrate buffer (pH 6.4) for 15 min. Tissue was permeablised with Triton-X-100 1% in PBS and blocked with BSA 0.2% in PBS for 1 h. Anti-*E. coli* (Europa Bioproducts, Cambridge, UK) 1∶2000, anti-ezrin (BD Biosciences, Oxford, UK) 1∶500 and anti-Ki67 (DAKO, Glostrup, Denmark) 1∶1000 primary antibodies were diluted in blocking solution and incubated with sections for 45 minutes–2 h at room temperature or overnight at 4°C. Alexa-fluor 488 and 594 fluorescent secondary antibodies were diluted 1∶2000 in PBS and incubated with sections for 30 minutes at room temperature. Sections were mounted with Vectasheid mounting medium with DAPI (Vector Laboratories, Cambridge, UK). In *ex vivo* tissue the possibility of cross staining endogenous *E. coli* was excluded by ensuring that matched, adjacent tissue (from the same patient) was completely negative for *E. coli* via immunofluorescence.

### Image capture & Quantitative image analysis

Fluorescent images were captured using either a Zeiss Axioskop 20 fluorescence microscope (Zeiss, Luton, UK) using Smartcapture software (Digital Scientific, Cambridge, UK) or a Zeiss Axioplan II fluorescence microscope (Zeiss, Luton, UK) using IPLab Spectrum software (Scanalytics Corp, Fairfax, VA). Image analysis was performed using in-house scripts written for IPLab. Briefly, software calculated the nuclear staining intensities for the proteins of interest in individual cells. The individual intensity values for over 300 cells were counted for each experimental condition. The entire experiment was repeated on three separate occasions. For MSH2 and MLH1, cells with staining intensity values below 5% of the maximum intensity (measured in uninfected controls) were categorized as MMR protein deficient. This is a categorization reflects the fact that low MMR protein expression levels (<10%) are required for a deficiency in MMR function [28]. The same categorization (i.e. above or below 5% of maximum intensity) was arbitrarily applied to PCNA staining.

## Results

### EPEC infection caused downregulation of DNA mismatch repair proteins in vitro

**Figure 1:**
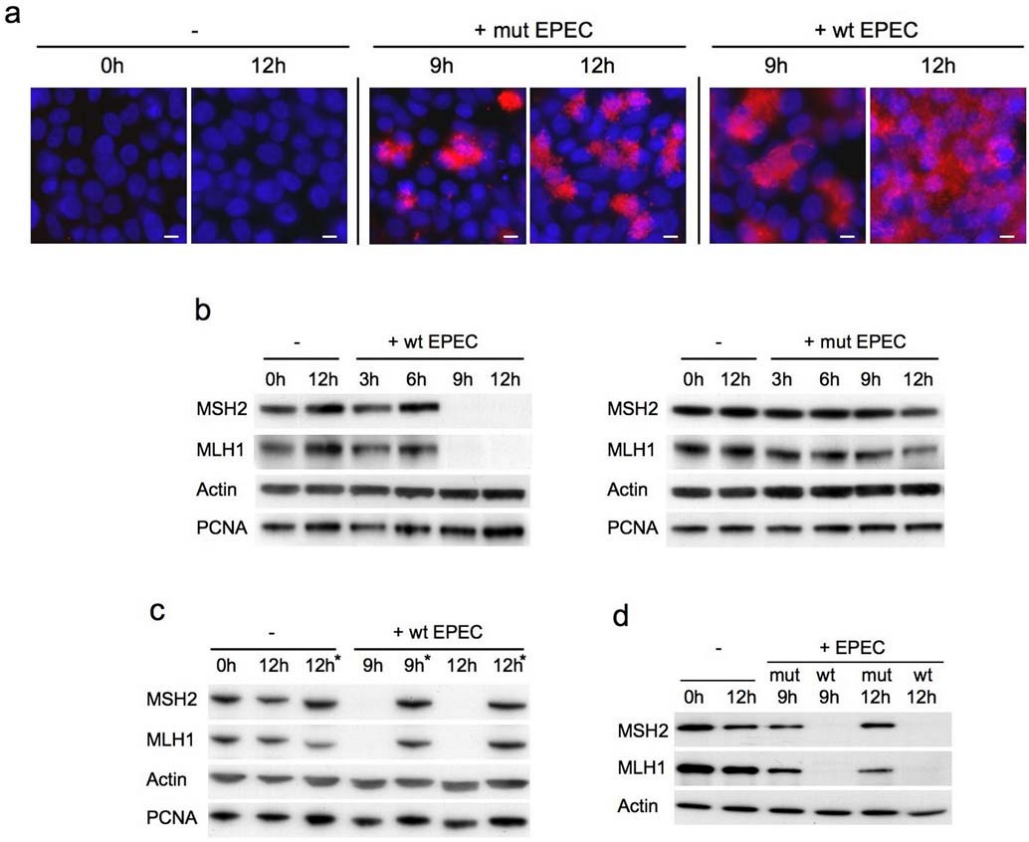
Co-culture of human colorectal cell lines with EPEC *in vitro*. Immunofluorescence staining for *E. coli* (red) and DAPI nuclear staining (blue) shows the adherence patterns of wild-type (E2468/69) and mutant (UMD864) EPEC (*a*). Immunoblot analysis of DNA mismatch repair protein expression in human colorectal cancer cell lines co-cultured with EPEC: HT29 cells were either uninfected (−) or co-cultured with wild-type (+wt) or mutant (+mut) EPEC for 3–12 hours (*b*). HT29 cells were either uninfected or co-cultured with wild-type EPEC for 9 or 12 hours then treated with antibiotics for 36–39 hours (*) to remove infection (*c*). SW480 cells were either uninfected or co-cultured with wild-type or mutant EPEC for 9 or 12 hours (*d*). EPEC strain UMD864 does not express EspB, a protein required for a functional type III secretion system. Scale bar = 10 µm.

To investigate the effects of prolonged EPEC infection on MMR protein expression, we established an extended time-course co-culture model. During the course of *in vitro* co-culture experiments EPEC causes cultured cells to detach from culture vessels by disrupting focal adhesions, adherens junctions and tight junctions [29]–[31]. Hence, EPEC induced loss of adhesion fundamentally limits the time-course of co-culture experiments. By using confluent cell monolayers and washing cells every 3 hours (to remove excess non-adherent bacteria) we were able to extend the co-culture time-course to 12 hours. Although some cell detachment did occur during this time-course, the majority of cells remained adherent at 12 hours; beyond this time-point loss of adherence made co-culture impractical. Figure 1a demonstrates the adherence patterns of wild-type and mutant EPEC after *in vitro* co-culture. Wild-type EPEC attach intimately to host cells and show widespread distribution across the cell monolayer after 12 hours. The mutant EPEC (with an internal deletion in the gene encoding EspB, a key component of the type III bacterial secretion system) are unable to attach intimately, but adhere in localised clusters using bundle forming pili.

Immunoblots revealed a marked reduction in MSH2 and MLH1 protein expression (relative to actin and PCNA which were both used as loading controls) in HT29 cells after 9–12 hours co-culture with wild-type EPEC. In contrast, uninfected cells and cells infected with non-intimately attaching mutant EPEC retained strong MMR protein expression (Figure 1b). Treating cells that had been infected with wild-type EPEC for 9–12 hours with antibiotics allowed them to recover from infection and survive long term. After only 36–39 hours antibiotic treatment, infected cells were able to recover MMR protein expression to pre-infection levels (Figure 1c). SW480 cells showed a similar reduction on MSH2 and MLH1 in response to EPEC (Figure 1d), indicating that this effect was not limited to a single cell line.

**Figure 2:**
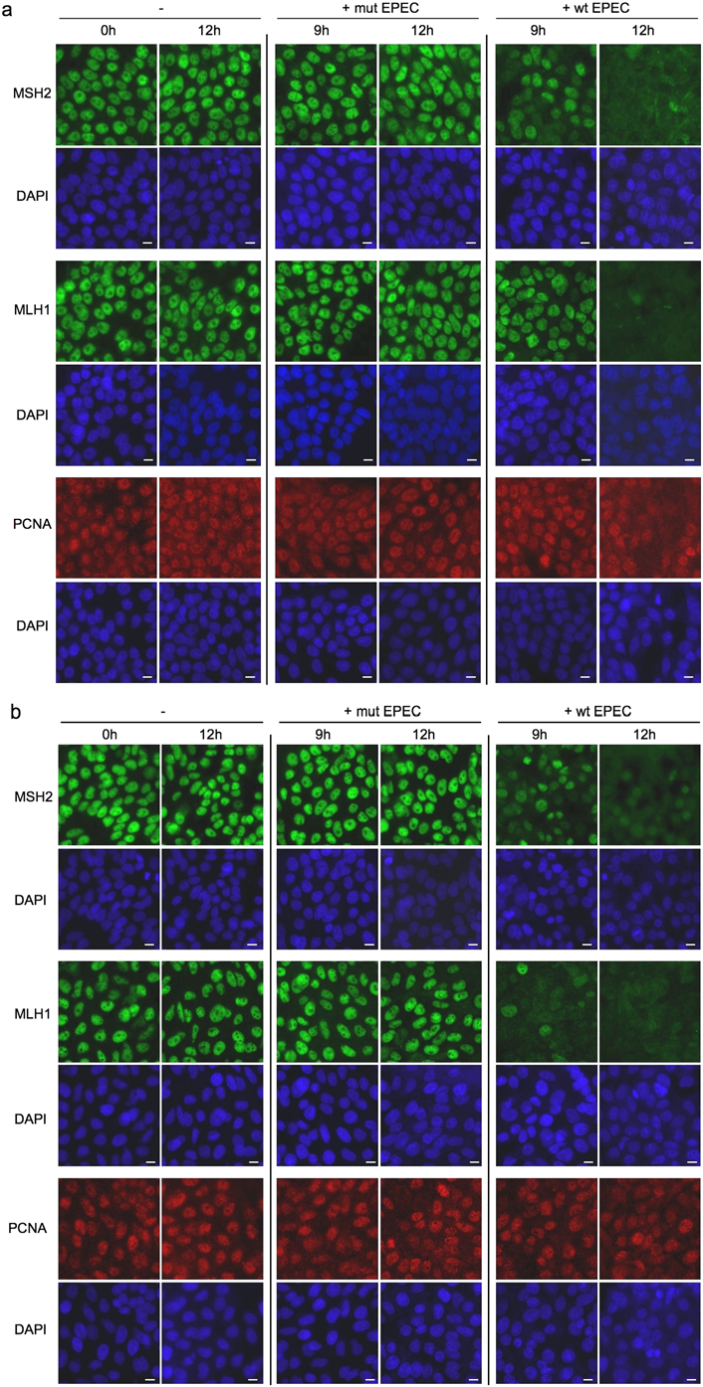
Immunofluorescence staining of DNA mismatch repair proteins in human colorectal cancer cell lines co-cultured with EPEC. HT29 (*a*) and SW480 cells (*b*) were either uninfected (−) or co-cultured with wild-type (+wt) or mutant (+mut) EPEC for 9–12 hours. Fixed cells were probed for MSH2, MLH1 (both green) and PCNA (red) by immunofluorescence, nuclei were counterstained with DAPI (blue). Scale bar = 10 µm.

**Figure 3:**
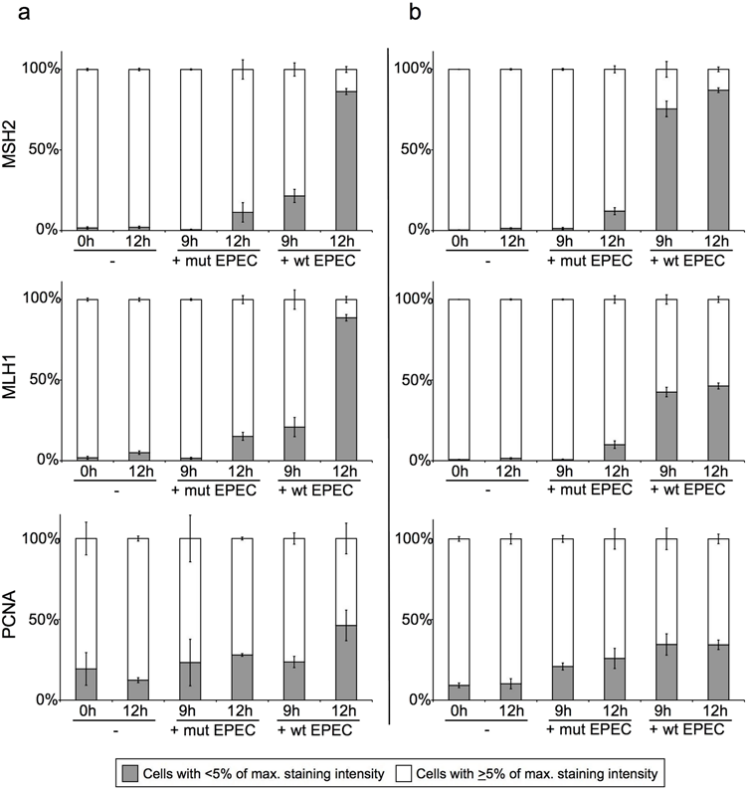
Quantitative image analysis of DNA mismatch repair protein immunostaining in human colorectal cancer cell lines co-cultured with EPEC. HT29 (*a*) and SW480 cells (*b*) were either uninfected (−) or co-cultured with wild-type (+wt) or mutant (+mut) EPEC for 9–12 hours. Fixed cells were probed for MSH2, MLH1 and PCNA by immunofluorescence. Digital images were quantitatively analysed for nuclear staining intensity of the proteins of interest. According to staining intensity values, individual cells were categorised as having an intensity value greater than or equal to 5% of the maximum staining intensity (as measured in uninfected controls) or less than 5% of the maximum staining intensity. Cells with MSH2 or MLH1 staining below 5% of the maximum were classed as MMR protein deficient. The entire experiment was performed on three separate occasions for each cell line, error bars represent standard error of mean.

As an alternative method to quantify the loss of MMR protein expression, we performed immunofluorescence staining combined with quantitative image analysis. Images of immunofluorescent stained uninfected and mutant infected HT29 and SW480 cells show that MSH2 and MLH1 expression was strong and localised to the nuclei. After 9 hours infection with wild-type EPEC, MSH2 and MLH1 expression was markedly reduced in some cells but retained in others. After 12 hours infection with wild-type EPEC the majority of cells had very low MMR protein expression. PCNA staining was used as a positive control for image analysis of nuclear staining. In comparison to MMR proteins levels PCNA expression levels remained relatively constant in the presence of EPEC infection (Figure 2a & b). Quantitative image analysis of immunofluorescence stained HT29 and SW480 cells confirmed that wild-type EPEC infection induced a marked reduction in MSH2 and MLH1 expression (Figure 3a & b). Image analysis graphs show that up to 90% of HT29 and SW480 cells were MMR protein deficient after 12 hours infection with wild-type EPEC. MMR protein downregulation was more rapid in SW480 cells (i.e. more MMR protein deficient cells present after 9 hours infection), but more complete in HT29 cells after 12 hours infection. Infection with the non-attaching and effacing mutant EPEC had very little effect on MMR protein expression after 9 hours, with a small increase in the number of MMR deficient cells after 12 hours. This demonstrates that MMR protein downregulation was predominantly dependent on translocation of effector proteins from bacteria to host cell and/or signalling due to intimate attachment. PCNA was used as a positive control for image analysis and its expression remained relatively constant in comparison with the MMR proteins.

### EPEC induced mismatch repair protein downregulation was not associated with apoptosis/necrosis

**Figure 4:**
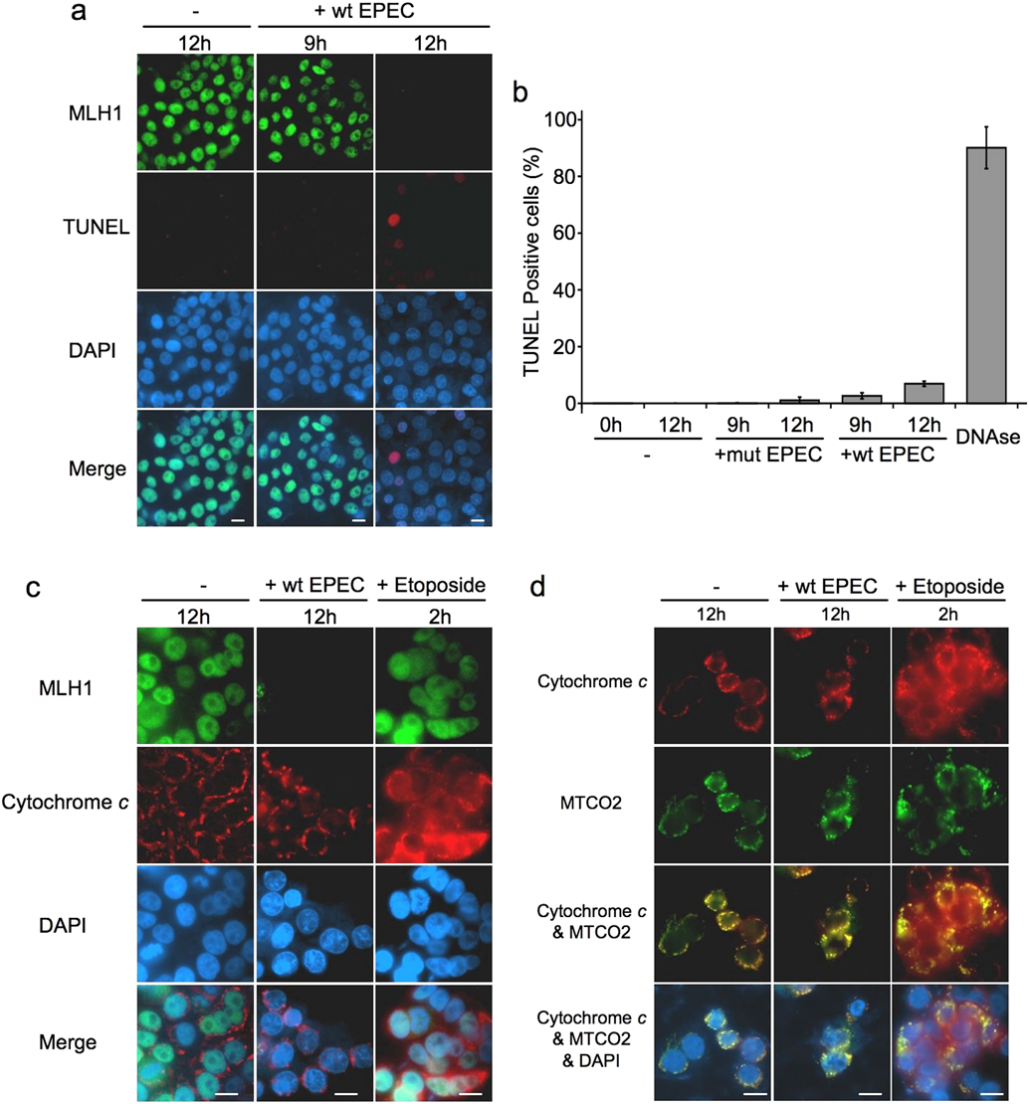
EPEC induced mismatch repair protein downregulation was not associated with apoptosis/necrosis. HT29 cells either uninfected (−) or co-cultured with wild-type EPEC (+wt) for 9–12 hours were stained (red) using an *in vitro* cell death (TUNEL) detection kit, MLH1 was simultaneously stained (green) and nuclei were counterstained with DAPI (blue) (*a*). HT29 cells were either uninfected (−) or co-cultured with wild-type (+wt) or mutant (+mut) EPEC; TUNEL positive and negative cells were counted. Fixed HT29 cells treated with DNAase (to induce DNA strand breaks) were used as positive controls, error bars represent standard error of mean (n = 3) (*b*). HT29 cells were stained for MLH1 (green) and cytochrome *c* (red) (*c*) or for cytochrome *c* (red) and the mitochondrial marker MTCO2 (green) (*d*). Cells treated with etoposide for 2 h were positive controls for early apoptosis. Scale bar = 10 µm.

Activation of protease enzymes during apoptosis results in the degradation of many intracellular proteins, including DNA MMR proteins [32]. We therefore investigated whether the effects of EPEC on MMR protein expression were related to apoptosis or necrosis. TUNEL staining demonstrated that most cells displaying EPEC induced MMR protein (MLH1) downregulation had not entered late stage apoptosis/necrosis (Figure 4a). Counts of TUNEL positive and negative cells show that although wild-type (and to a lesser degree mutant) EPEC infection caused an increase in the number of cells undergoing apoptosis/necrosis, this change was relatively small compared with the number of cells showing MMR protein downregulation (Figure 4b).

It was possible that infected cells had not had sufficient time to reach late stage apoptosis as measured by the TUNEL assay, so to identify cells undergoing the early stages of apoptosis we analysed cytochrome *c* distribution [33]. In uninfected cells and cells infected with wild-type EPEC for 12 hours, cytochrome *c* was distributed in discrete cytoplasmic regions (mitochondria). In cells treated with etoposide as a positive control, cytochrome *c* distribution was diffuse. This demonstrates that the release of cytochrome *c* from mitochondria was occurring in these cells, an indicator of commitment to the early stages of apoptosis. In addition, MLH1 expression was strong in both uninfected and etoposide treated cells, but absent in cells infected with EPEC for 12 hours (Figure 4c). Cytochrome *c* distribution was confirmed to be limited to the mitochondria in both uninfected and EPEC infected cells (but not in etoposide treated cells) by staining mitochondrial marker MTCO2 (Figure 4d). Cytochrome *c* staining was also performed on cells infected with wild-type EPEC for 9 hours and showed the same distribution as in uninfected cells and cells infected for 12 hours (not shown).

### Co-culture of ex vivo adult human colonic mucosa with EPEC

EPEC is the most prevalent form of AEEC found in man, EPEC has been reported to adhere to adult colon tissue [34], but it has not been previously reported whether EPEC are capable of entering colonic crypts. Colonic tumours are thought to develop from proliferative epithelial cells found within colonic crypts [35]. We wished to investigate whether EPEC could enter colonic crypts and attach to cells in the proliferative compartment. Normal colonic mucosa was removed from three adults; mucosal strips from each patient were either uninfected or co-cultured with wild-type EPEC for 12 hours. Fixed mucosa sections were probed by immunofluorescence and the number of crypts containing *E. coli* was counted. In total, *E. coli* were detected in 195 of 1928 (10.1%, range 4.8%–14.2%) crypts in tissue that had been co-cultured with EPEC. In contrast, no *E. coli* were found in 1797 crypts examined from uninfected tissue (P<0.001, chi square test, two tails). It was noted that *E. coli* did not show widespread adherence to the surface epithelium of the mucosa (i.e. outside the crypts). Within individual crypts the number of bacteria (per 3 µm cross section) varied from single cells to colonies of tens and in some cases up to approximately one hundred bacteria. Successive sections demonstrated that bacterial colonies spread along the width as well as the length of the crypts. Crypts that were positive for *E. coli* were distributed in a random fashion throughout the tissue cross-section and did not therefore occur due to lateral infection.

**Figure 5:**
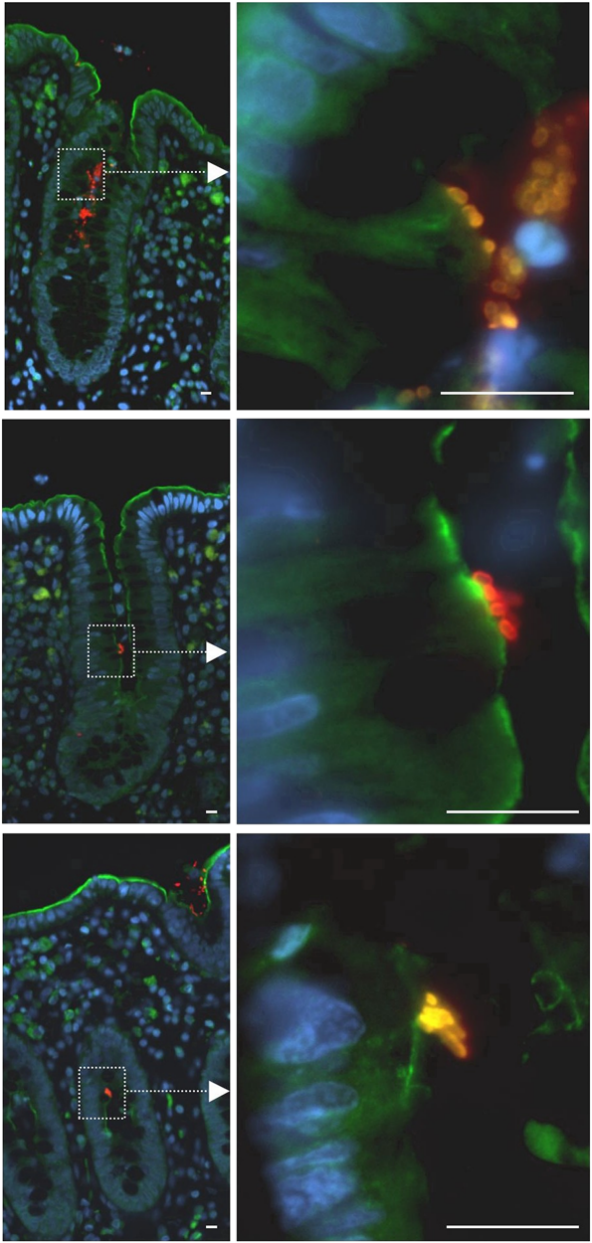
Co-culture of *ex vivo* adult human colonic mucosa with EPEC. Sections of colonic mucosa were removed from surgical patients and co-cultured with wild-type EPEC for 12 hours, then fixed in buffered formalin and embedded in paraffin. Images show examples of tissue sections stained by immunofluorescence for *E. coli* (red) and ezrin (green) as a marker of the cell surface and DAPI (blue). Scale bar = 10 µm.

To assess whether EPEC were closely associated with the apical surface of crypt epithelial cells we stained mucosa sections with ezrin. Ezrin is a structural protein that anchors the cytoskeleton to the plasma membrane and is found at sites of EPEC induced AE lesions [36]. Staining of ezrin confirmed that EPEC was often closely associated with (and potentially intimately attached to) the apical surface of crypt epithelial cells (Figure 5). It can also be seen from Figure 5 that EPEC were found in the mid-region of the crypts. This distribution overlaps with the proliferative region of the crypts [37]. These results demonstrate for the first time that EPEC are able to enter crypts of the adult human colon, penetrate into the proliferative compartment and associate with crypt epithelial cells.

### Analysis of human colorectal adenocarcinomas for mucosa-associated *E. coli* and attaching and effacing *E. coli*

**Figure 6:**
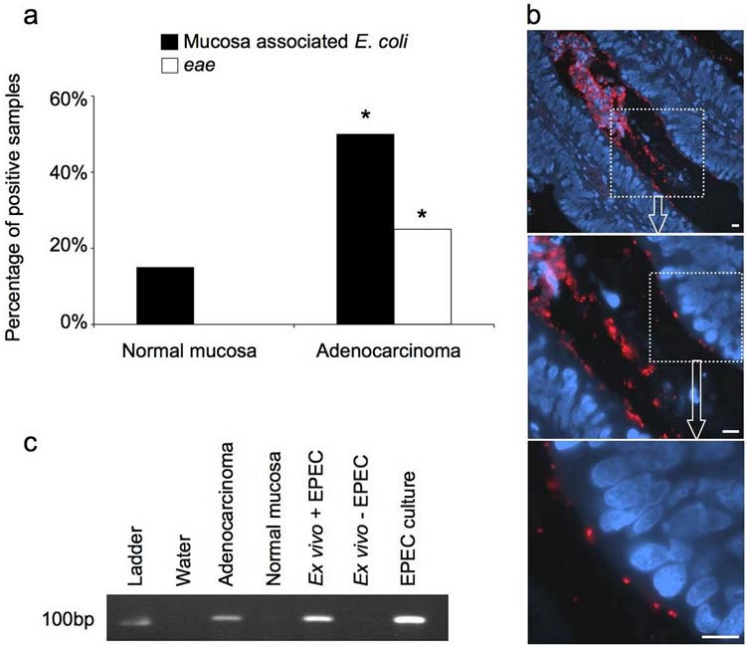
Analysis of human colorectal adenocarcinomas and normal colorectal tissue for attaching and effacing *E. coli*. Paraffin embedded sections of adenocarcinoma and matched normal tissue from 20 patients were probed for *E. coli* by immunofluorescence and for the bacterial gene *eae* by PCR (*P<0.05, Fisher's Exact Test, two tails) (*a*). Example of an adenocarcinoma with mucosa associated *E. coli* (red), nuclei stained with DAPI (blue) (*b*) that tested positive for eae by PCR (*c*). Sections of *ex vivo* human colonic mucosa that were either co-cultured with (+) or without (−) wild-type EPEC were used as additional controls for PCR. Scale bar = 10 µm.

Previous reports have demonstrated that *E. coli* are often found attached to colonic adenocarcinomas; we wished to investigate this association and assess whether AEEC strains were present. Matched normal colonic mucosa and colonic adenocarcinoma tissue from 20 patients was analysed for mucosa-associated *E. coli* (by immunofluorescence) and the presence of *eae* (by PCR). Mucosa-associated *E. coli* were identified in 3/20 samples (15%) of normal mucosa, 0/20 (0%) samples tested positive for *eae*, whereas 10/20 (50%) of adenocarcinomas were positive for mucosa-associated *E. coli*, 5/20 (25%) of which were *eae* positive (Figure 6a). The increased frequencies of mucosa-associated *E. coli* and AEEC on adenocarcinomas compared to normal tissue were statistically significant (P<0.05 for both comparisons, Fisher's Exact Test, two tails). Mucosa-associated *E. coli* were clearly visible on the apical surface of epithelial cells within tubulo-villus crypts of adenocarcinomas, as demonstrated by the example shown in Figure 6b. The number of individual *E. coli* per adenocarcinoma section ranged from tens to tens of thousands. Figure 6c is a representative PCR result, which shows that the adenocarcinoma shown in Figure 6b tested positive for *eae*, but that normal colonic mucosa from the same patient did not. Formalin fixed, paraffin embedded sections of human *ex vivo* colon mucosa that were either uninfected or infected with wild-type EPEC were used as additional negative and positive PCR controls respectively.

## Discussion

Previous studies have established that mucosally adherent *E. coli* are often present on colonic tissue from colorectal cancer patients [4], [5]. The results of the present study strongly support this association. In addition, this study demonstrates for the first time an association of AEEC strains with human colorectal adenocarcinomas. Furthermore, we uncovered a mechanism by which AEEC might contribute to the development of colorectal carcinoma by downregulating mismatch repair proteins that protect cells against accumulating mutations. EPEC strains are the predominant AEEC strains identified in human subjects [8]–[10]. As EPEC screening is no longer performed in developed countries there is currently a lack of data on the carriage of EPEC among the healthy adult population of these countries. Presently, studies on the prevalence of EPEC infection focus on children, and show that up to 10.9% of asymptomatic (i.e. healthy) children in developed countries carry EPEC [10]. In the sample of colorectal cancer patients we tested, AEEC were identified in 25% of cancer tissue specimens, but in no specimens of normal tissue.

A limitation of the present study is the relatively small number of patients tested (n = 20), resulting in wide confidence intervals around this estimate (6% and 43%). Despite this limitation, the result was statistically significant and suggests that a substantial percentage of adults with colorectal cancer carry AEEC. Studies on additional populations of cancer patients (along with healthy controls) are required to substantiate this association. Another limitation was the use of paraffin sections, which made it impossible to isolate bacteria from tissue samples. Future studies of cancer patients should therefore utilise fresh tissue, from which the specific bacterial strains, serotypes, clonal lineages and virulence factors could be identified.

Though we have identified an association between AEEC and colorectal cancer, it is presently unknown if this association is causal. The difference in AEEC distribution between normal and adenocarcinoma tissue may be due to the affinity of EPEC for surface residues on tumour cells. Surface antigens of fetal and tumour cells display some homology, leading to the suggestion by Martin *et al.* (2004) that adherent *E. coli* bind to ‘oncofetal’ surface antigens created by altered mucosal glycosylation [4]. In addition to improved binding affinity, the increased surface area of tumour versus normal tissue also facilitates binding of far greater numbers of adherent bacteria. This observation could also explain why tumourigenesis may be of benefit to the bacteria, as tumours seemingly provide an environment conducive to bacterial adhesion and growth.

Previous studies that have employed co-culture of EPEC with human intestinal tissue generally focus on bacterial adhesion within the small intestine and use tissue removed from children [26], [38]–[40]. Results of a recent study show that EPEC strains are able to adhere to adult colon tissue but the culture method employed did not reveal where the bacteria were bound [34]. A key aspect of the present study was therefore to identify the sites of EPEC adhesion within the adult colonic mucosa and specifically whether EPEC entered colonic crypts. We found that EPEC readily entered colonic crypts and attached to epithelial cells, often in the mid-region of the crypts. The mid and lower sections of colonic crypts contain undifferentiated proliferative epithelial cells. Colorectal cancer is thought to arise from transformation of these cells [35]. The washing procedures used during co-culture are likely to have reduced the levels of surface mucus, potentially facilitating the adhesion of bacteria. However, it was macroscopically apparent that mucus was secreted from the *ex vivo* mucosa throughout the co-culture time-course, and immunosections illustrate that crypt goblet cells contain mucus after 12 hours co-culture. EPEC were able to enter the crypts and attach to epithelial cells despite this.

A potential explanation for the apparent affinity of EPEC for crypt cells is the change in expression of surface glycoproteins during cell migration from crypt to surface epithelium [41], [42]. Within crypts, mitotically active, undifferentiated cells (in similarity to fetal/cancer cells) express active glycosyltransferase enzymes and acceptor sites that are glycoproteins with incomplete polysaccharide chains [43], [44]. A selective ability to bind to the surface residues of these undifferentiated cells could explain the affinity of EPEC for adenocarcinoma cells and the more severe pathology of EPEC infection in the infant intestine. The abilities of EPEC to enter crypts of the adult human colon and attach to crypt epithelial cells are novel findings, and illustrate a potential niche environment for EPEC in the adult colon that requires further investigation.

Intimate attachment of EPEC to epithelial cells involves injection of bacterial proteins into the host cell, resulting in up and down regulated expression of a wide range of host cell proteins [20]. We hypothesised that the effects of EPEC infection on the host cell could promote susceptibility to cancer. One candidate target for disruption is DNA mismatch repair (MMR), a system that maintains genomic stability by correcting DNA base pair mismatches. Inactivating mutations of the DNA MMR genes *MSH2* and *MLH1* are a causative genetic feature of many colorectal cancers.

In co-culture experiments with colorectal cell lines we observed that wild-type EPEC caused a dramatic reduction in the expression of the key MMR proteins MSH2 and MLH1. Despite causing a marked downregulation of these proteins, wild type EPEC infection was not associated with widespread induction of apoptotic/necrotic mechanisms. This is a logical finding since MMR proteins are involved in signalling DNA damage induced apoptosis, deficiency of these proteins therefore suppresses apoptosis [45]. We also demonstrated that cells infected for up to 12 hours with wild-type EPEC were able to survive after removal of infection. DNA replication in the absence of functional DNA mismatch repair and DNA damage associated apoptotic signalling would present an environment highly conducive to the accumulation of mutations.

In mouse models, lack of functional DNA MMR causes increased spontaneous mutation frequency rates in all parts of the colon [46] and enhances somatic mutation of *Apc* and *p53*; genes mutated in the majority of colorectal cancers [47]. It is therefore possible according to our current hypothesis, that EPEC infection could cause a transient mismatch repair inactivation that may drive carcinogenesis by promoting somatic mutations. Cancers evolving through this hypothetical pathway may therefore present with some degree of microsatellite instability or microsatellite alterations in cancer relevant genes (e.g. *APC*, *p53*) even if they are not categorised as being microsatellite unstable. It is notable that some microsatellite stable colorectal tumours do indeed present with frameshift mutations in repetitive regions of *APC* [48]. Whether EPEC infection could be responsible for promoting such mutations remains to be elucidated - analysing this subset of sporadic tumours for EPEC infection could help to answer this question, though clearly an observed EPEC infection may be independent of (and consequent to) the carcinogenic event. More directly, probing tumours infected with EPEC for MSI would help to establish whether or not infection could promote this genetic aberration.

It should be noted that MMR failure also promotes mutations in non-repetitive regions of DNA (in addition to microsatellite sequences), as shown by studies on mutation spectra in human cells [49]–[52]. Analysis of mutation spectra in yeast also showed that MMR mutants (*msh2*, *msh6*, *mlh1* and *pms1*) had increased frameshift mutation rates in non-repetitive regions compared to wild-type [53]. Microsatellite instability is not therefore the sole result or indicator of DNA MMR failure, and transient MMR inactivation (e.g. via EPEC infection) could promote mutations in cancer relevant genes without causing MSI.

This study identifies a striking similarity between EPEC and the carcinogenic bacterium *Helicobacter pylori*, which has also been found to downregulate MSH2 and MLH1 expression in human cell lines [54]. Although not classed as an AE bacterium, *H. pylori* attach to and interact intimately with gastric epithelial cells [55], [56] similarity to EPEC, some *H. pylori* strains also have a secretion system used to translocate effector proteins (such as CagA) into host cells [57]. It is notable that *H. pylori* strains that carry the *cag* pathogenicity island (required for the bacterial type-IV secretion system) have increased ability to cause cancer versus *cag* negative strains [58].

Quantitative immunofluorescence demonstrated that MMR protein downregulation occurred more rapidly in SW480 cells versus HT29 cells. SW480 cells show less features of differentiation (e.g. enterocytic differentiation, cell polarity) than HT29 cells [59]. We speculate that the reason EPEC is able to initiate its effects more rapidly in SW480 cells may be because it can bind more efficiently to undifferentiated cells. This property would help to explain the apparent binding affinity of EPEC for cells that are ‘less differentiated’ *in vivo* i.e. adenocarcinoma, fetal and possibly proliferative crypt cells. It is logical that EPEC would target more immature cells when invading a host. Firstly, these cells are likely to live for longer than differentiated cells (especially in the colon where cell turnover is rapid), providing a longer lasting binding site. Secondly, it is possible that the molecular and cellular consequences of EPEC infection are more easily effected in undifferentiated cells, where cell fate is yet to be determined.

In the present study we have characterised and quantified the effect of EPEC infection on DNA MMR proteins expression *in vitro*. The exact mechanisms responsible for this effect were beyond the remit of the present study and require further investigation. We have demonstrated that a mutant EPEC strain (lacking EspB, a key component of the bacterial type-III secretion system) did not induce significant MMR protein downregulation. This indicates that EPEC induced MMR downregulation was dependent on translocation of bacterial effector proteins and/or intimate attachment. EPEC secreted effector proteins have been shown to target specific host proteins and organelles; e.g. EspF targets tight junction proteins and mitochondria [31], [60]. The time-scale of EPEC induced MMR protein downregulation suggests that MMR protein degradation may be occurring in the host cell. Degradation of MMR proteins can occur via proteosomal degradation subsequent to ubiquitination [61], a process that may be targeted by effector proteins. Co-culture experiments with a panel of mutant EPEC strains (with specific bacterial effectors knocked out) would help uncover the effector protein(s) responsible for effects on MMR. It will also be important to investigate whether other AEEC strains are able to induce MMR protein downregulation. Of particular interest are atypical EPEC strains, which are of increasing importance in developed countries.

Regulation of MMR protein can occur at transcriptional level by a number of pathways and it is possible that transcriptional silencing also contributes to EPEC induced MMR protein downregulation. Microarray analysis of HT29 cells revealed a 2–4 fold reduction in RNA levels of mismatch repair genes *MSH2*, *MLH1* and *MSH6* in wild-type EPEC infected cells (but not *espB* mutant EPEC infected cells) versus uninfected controls (unpublished data). In cancer cell lines, suppression of DNA MMR has been observed via inhibition of E2F transcriptional activity by Bcl-2 [62]. Bcl-2 function is regulated by phosphorylation, mediated by Protein Kinase Cα (PKCα) [63]. EPEC infection increases membrane bound PKCα activity and the PKCα content of cancer cell membranes [64]. This suggests that EPEC-induced activation of PKCα might result in phosphorylation of Bcl-2, thus promoting cell survival and inhibiting the transcription of MMR genes. EPEC induced MMR protein downregulation provides a mechanism by which infected cells could have a significantly increased susceptibility to mutation. The consequences of EPEC induced MMR protein downregulation on spontaneous and induced mutation frequency rates therefore requires investigation.

The findings of this study suggest that chronic EPEC infection in asymptomatic adults could promote tumourigenesis within the colon by influencing the molecular biology of colonic epithelial cells. In support of this idea, we have identified a high incidence of AEEC in patients with colonic adenocarcinoma and an association between AEEC and tumours in these patients. The role of EPEC in colonic tumourigenesis therefore warrants further investigation; of particular interest is the prevalence of EPEC infection in healthy adults versus those with colonic adenomas and carcinomas.
